# Molecular Characterization of a Tetraspanin TSP11 Gene in *Echinococcus granulosus* and Evaluation Its Immunoprotection in Model Dogs

**DOI:** 10.3389/fvets.2021.759283

**Published:** 2021-11-17

**Authors:** Jinwen Xian, Pengpeng Zhao, Ning Wang, Weiye Wang, Yanyan Zhang, Jimeng Meng, Xun Ma, Zhengrong Wang, Xinwen Bo

**Affiliations:** ^1^State Key Laboratory of Sheep Genetic Improvement and Healthy Production/Institute of Animal Husbandry and Veterinary Medicine, Xinjiang Academy of Agricultural and Reclamation Sciences, Shihezi, China; ^2^College of Animal Science and Technology, Shihezi University, Shihezi, China

**Keywords:** *Echinococcus granulosus*, cystic echinococcosis, Eg-TSP11, immunogenicity, dog vaccine

## Abstract

Cystic echinococcosis (CE) is a cosmopolitan zoonosis caused by the larval stage of *Echinococcus granulosus*, which affects humans and a wide range of mammalian intermediate hosts. Parasite tetraspanin proteins are crucial for host-parasite interactions, and therefore they may be useful for vaccine development or disease diagnosis. In the present study, the major antigen coding sequence of tetraspanin 11 (Eg-TSP11) from *E. granulosus* was determined. The results of immunolocalization showed that Eg-TSP11 was mainly located in the tegument of adult worms and protoscoleces. Western blotting analysis showed that the serum from dogs injected with recombinant Eg-TSP11 (rEg-TSP11) could recognize Eg-TSP11 among natural protoscolex proteins. Moreover, the serum from dogs with *E. granulosus* infection also recognized rEg-TSP11. Serum indirect enzyme-linked immunosorbent assays demonstrated that IgG levels gradually increased after the first immunization with rEg-TSP11 compared with those in the control group. Furthermore, the serum levels of interleukin 4, interleukin 5, and interferon gamma were significantly altered in the rEg-TSP11 group. Importantly, we found that vaccination with rEg-TSP11 significantly decreased worm burden and inhibited segment development in a dog model of *E. granulosus* infection. Based on these findings, we speculated that rEg-TSP11 might be a potential candidate vaccine antigen against *E. granulosus* infection in dogs.

## Introduction

Cystic echinococcosis (CE) is a serious zoonotic parasitic disease caused by *Echinococcus granulosus* larvae. CE is a major public health concern in developing and developed countries (1, 2). In epidemic areas, the incidence of CE is between 1 and 200 per 100,000 (3, 4). CE seriously endangers human health and the development of animal husbandry and causes the loss of at least 285,500 disability adjusted life years (DALYs) each year (5, 6). The disease is a neglected tropical disease, confirmed by the World Health Organization (WHO) (2). To date, the most successful intermediate host vaccine is the EG95 recombinant protein, with a protective efficiency of up to 98% (7). However, the recombinant EG95 protein had no protective effect on hosts have been infected with *E. granulosus* and the cysts have been formed. Canids, such as dogs, are the definitive hosts of *E. granulosus*, which play an important role in the life cycle of *E. granulosus*. Unfortunately, there is no commercially available definitive-host vaccine, which has seriously hindered the effective control of CE. Therefore, the development of a dog vaccine against *E. granulosus* is urgently required.

The tetraspanin superfamily (TSP, also known as the transmembrane-4-superfamily, TM4SF) is a hydrophobic plasma membrane-associated protein of 200–350 amino acids. Tetraspanins can be divided into four families, including the cluster of differentiation (CD) family (e.g., CD9, CD81, and CD151), the retinal degeneration slow (RDS) family (e.g., RDS-ROM), the uroplakin family (e.g., UPK1A/1B), and the CD63 family (e.g., CD63 and TSPAN31) (8). The four conserved transmembrane domains of TSPs are called tetraspanin-enriched microdomains (TEMs). TEMs protrude 3–5 nm from the cell surface to form two extracellular rings, one large and one small (EC1 and EC2). In addition, two short N-and C-terminal cytoplasmic tails are formed in the intracellular region. The large extracellular loop (LEL), which contains 2–6 cysteines, is called the “tetraspanin web” which plays a central role in the interaction of TSPs with several other molecules (9, 10).

To date, marked progress has been made in research on tetraspanins as parasite vaccine candidates. Tetraspanin family proteins now occupy an important position in parasite immune interactions, and have been proven to be candidate target proteins for schistosomiasis, Clonorchis sinensis, alveolar echinococcosis, and filariasis (10–13). Tetraspanins are involved in trematode cuticle development, maturation, stabilization, and immune escape (14). The two hydrophilic groups (Sm-TSP-1 and Sm-TSP-2) of the recombinant tetraspanin of *Schistosoma mansoni*, especially Sm-TSP-2, have been shown to be able to induce a >40% immunoprotective effect against *S. mansoni* infection (15). The *TSP1* gene of *E. granulosus* was cloned and expressed for the first time in 2015 and it was demonstrated that TSP1 could stimulate a marked Th1 type immune response in a mouse model. This means that TSP1 may be a possible target in the treatment, prevention, and control of echinococcosis (8). Tetraspanins play important roles in the study of the life cycle of parasites and in mediating parasite signal transduction, immune escape, and other important biological processes. As cell surface molecules, tetraspanins play important roles as bridges in parasite cell signaling pathways and thus represent potential therapeutic targets (9). The present study aimed to characterize the biological characteristics of TSP11 in *E. granulosus*. In addition, the immunoprotective effect of recombinant tetraspanin 11 Eg-TSP11 (rEg-TSP11) was analyzed by evaluating changes in the levels of IgG, and the cytokines Th1 and Th2 in model dogs. Furthermore, we analyzed the worm burden reduction rate and the inhibition of segment development in rEg-TSP11-vaccinated dogs. The results of the present study provide basic immunogenicity data for rEg-TSP11 and pave the way for the development of anti-*E. granulosus* vaccines in dogs.

## Materials and Methods

### Parasites

Naturally infected sheep livers, obtained from an abattoir in Urumqi, Xinjiang Province, China, were the source of hydatid cysts. Cyst fertility was checked using light microscopy to determine the presence of protoscoleces (PSCs) within the cysts. PSCs were isolated and treated according to previously described methods (16). PSCs (*n* = 2,000) were grown in 1 mL Roswell Park Memorial Institute (RPMI) 1640 medium containing 100 μg/mL streptomycin and 100 U/mL penicillin (Sigma-Aldrich, St. Louis, MO USA) and 10% bovine serum albumin (BSA; Hyclone, Logan, UT, USA). At 28 days after artificial PSC infection in an 8-month-old dog, adult worms were obtained.

### Bioinformatic Analysis

The cDNA sequence of Eg-TSP11 (XP_024352489.1) was downloaded from the NCBI database. The physicochemical parameters of the encoded proteins were analyzed using ProtParam tools on the ExPASY website (https://web.expasy.org/protparam/). SignalP 4.1 (http://www.cbs.dtu.dk/services/SignalP/) was used to check for the presence of a signal peptide, and Novopro tools were used to analyze the transmembrane regions (https://www.novopro.cn/tools/tmhmm.html). SWISS-MODEL (http://swissmodel.expas y.org/) was used to model the tertiary (three-dimensional) structures. The B cell epitopes of Eg-TSP11 were predicted using the IEDB online server (http://tools.immuneepitope.org/main/). MEGA software (version 5.05) used the neighbor-joining (NJ) method to construct phylogenetic trees of aligned proteins (17).

### Expression and Purification of the Eg-TSP11 LEL Region

An RNA-prep Pure Tissue Kit (Nanjing Vazyme Biotech, Nanjing, China) was used to extract total RNA from the PSCs. First-strand cDNA was synthesized from total RNA using a reverse transcription system kit (Nanjing Vazyme Biotech). PCR was then used to amplify the LEL coding sequence from the cDNA using a sense primer (5′- CGC GGA TCC ATG TTT CCA GCA CCG CTT CAA G-3′) comprising a *BamHI* site (underlined) and an antisense primer (5′-CCG CTC GAG TCA TTC ATA GTT TTT CAA GGA G-3′) comprising a *XhoL I* site (underlined). The PCR amplicons were ligated into the pET32a (+) plasmid (Novagen, Darmstadt, Germany) and transformed into *Escherichia coli* BL21 (DE3) cells (Tiangen, Beijing, China). Isopropyl-1-thio-β-D-galactopyranoside (1 mM; IPTG) was used to induce expression from the plasmid for 6 h at 37°C. Inclusion bodies were obtained from the *E. coli* cells, suspended in lysis buffer containing 8 M urea, and incubated for 2.5 h on ice to completely solubilize the recombinant protein. Ni^2+^ affinity chromatography with a His-affinity resin column (Bio-Rad, Hercules, CA, United States) was used to purify the His-tagged rEg-TSP11 protein, which was subjected to 10% sodium dodecyl sulfate-polyacrylamide gel electrophoresis (SDS-PAGE). A NanoDrop 2000c (Bio-Rad) instrument was used to determine protein concentration.

### Western Blotting

SDS-PAGE (10%) was used to separate rEg-TSP11 and total proteins in extracts from PSCs. The separated proteins were then electrotransferred onto nitrocellulose membranes. Membranes were blocked with 5% (w/v) skim milk for 2 h at 37°C. The membranes were then incubated with *E. granulosus* positive or negative dog sera, anti rEg-TSP11 dog sera, or pre-immunized mouse sera (1:100 v/v dilutions) at 4°C overnight. The next day, the membranes were washed and incubated with horseradish peroxidase (HRP)-conjugated rabbit anti-dog IgG or sheep anti-mouse IgG (1:5,000 v/v dilution) for 2 h. Actin protein of *E. granulosus* was used as an internal control. The membranes were incubated with Pierce ECL western blotting substrate (Thermo Fisher Scientific, Waltham, MA, USA) and then exposed to X-ray films.

### Quantitative Real-Time Reverse Transcription PCR

Quantitative real-time reverse transcription PCR (qRT-PCR) was used to examine the expression profiles of Eg-TSP11 in 28-day strobilated worms and PSCs. Total RNA was isolated from 28-day strobilated worms and PSCs, and cDNA was synthesized from total RNA. The cDNA was then used as a template for qPCR using the TSP11 primers: 5′- GAA GAT AAT GGC TGG GGT GC-3′ and 5′-GTT GTG TGC CCC ATT TGT GA-3′. Actin gene expression was used as an internal control for normalization. Primers for amplification of *E. granulosus* actin were 5′-GAG TCA TGT AGG CCA CG-3′ and 5′-AGA TGG AGG TGG GGA TAG G-3′. The 2^−ΔΔCT^ method was used to analyze the data (18).

### Immunolocalization

To identify the location of TSP11 at different developmental stages, adult worms and fresh PSCs were fixed overnight using 4% paraformaldehyde hydrophosphate buffer, permeabilized for 30 min using 1% Triton X-100, and incubated in 0.01% Triton X-100 at 4°C for 1 h. They were then washed three times with 0.01 × phosphate-buffered saline (PBS) and blocked with 5% (w/v) skim milk for 2 h at 37°C. Next, the fixed adult worms and PSCs were incubated with anti-rEg-TSP11 dog IgG (1:100 v/v dilution in PBST) at 4°C overnight. After washing, the sections were incubated with phycoerythrin (PE)-conjugated goat anti-dog IgG (H+L) (1:1,000 v/v dilutions in PBST) for 2 h in the dark at room temperature (25°C). The sections were washed four times with PBST and then examined under a fluorescence microscope (Leica, Wetzlar, Germany). The negative control was comprised of antibodies from the pre-immunized mice.

### Vaccination and *E. granulosus* Challenge

The vaccination experiment included nine Beagles. Group 1 was comprised of three dogs that were vaccinated with rEg-TSP11 mixed with the saponin adjuvant Quil A; Group II was comprised of three dogs that were vaccinated with Quil A only; and Group III was comprised of three dogs that were vaccinated with PBS (control group). Each 350 μL dose of vaccine included 200 μg of soluble rEg-TSP11 and 100 μg of Quil A (Sigma) in PBS. Before vaccination, the mixture was stirred overnight at 4°C. All experiments beagle dogs were immunized through subcutaneous injection in the neck and all the dogs were immunized with the same dose four times, at a 14 days interval. Seven days after the last booster vaccination, all nine dogs were challenged orally with 100,000 *E. granulosus* PSCs. Finally, at 28 days after infection (before eggs appeared), all nine dogs were euthanized and necropsied to collect and count worms as previously described (19, 20). Thirty worms were chosen randomly from each experimental group and the sizes of developed (≥4 segments) vs. underdeveloped (≤3 segments) worms were determined (21).

### Indirect Enzyme-Linked Immunosorbent Assay (ELISA)

The standard checkerboard titration procedure was used to assess the optimal concentrations of the rEg-TSP11 antigen and serum. Carbonate buffer (0.1 M, pH 9.6) was used to dilute the purified rEg-TSP11 protein to 5 μg/mL, which was used as the antigen in the ELISA test. The diluted antigen solution was used to coat ELISA plates at 4°C overnight. The next day, the plate was washed with PBS-Tween-20 (PBST) and then incubated with 5% skim milk at 37°C for 2 h. After thorough washing, 100 μL of serum samples (2-fold dilutions, 1:80) in PBST was added to each well and incubated for 1.5 h at 37°C. After washing, HRP-labeled rabbit anti-dog IgG (1:3,000 dilution; Solarbio, Beijing, China) was added to the plates and incubated for 1.5 h at 37°C. After washing, the substrate, 3, 3′, 5, 5′-tetramethylbenzidine (TMB) (Tiangen, Beijing, China) was added to the wells and incubated for 1.5 h at 37°C. Finally, 1 M H_2_SO_4_ (100 μL) was added to stop the development of color, and the optical density was measured at 450 nm (OD 450).

### Cytokine Detection

Five main cytokines in dogs with *E. granulosus* infection, including interferon gamma (IFN-γ), interleukin (IL)-6, IL-5, IL-4, and IL-1 were determined using an ELISA kit (Janglaibio, Shanghai, China). The required strip was removed from the aluminum foil bag after being left for 60 min at room temperature (25°C). The standard sample well, blank well, and sample well were set, and 50 μL of standard product at different concentrations was added. Then, HRP-labeled antibody (100 μL) was added to each well, which was sealed using a sealing film and incubated for 1 h at 37°C. The liquid was discarded, and the plate was patted dry using absorbent paper. Next, 350 μL of detergent was added to each well and incubated for 1 min, after which the detergent was shaken and the plate was patted dry using absorbent paper. The detergent wash step was repeated five times. Then, 50 μL of substrate A and 50 μL of substrate B were added to each well and incubated for 15 min at 37°C. Finally, 50 μL of termination solution was added to each well, and within 15 min, the OD value of each well was measured at a wavelength of 450 nm.

### Data Analysis

Statistical analyses were performed using SPSS software (version 22.0; IBM Corp, Armonk, NY, USA). All data analyses and graphs were performed using GraphPad Prism 6.0 software package (GraphPad Software Inc., San Diego, CA, USA). Statistical significance was set at *P* < 0.05. All experiments were repeated a minimum of three separate times.

## Results

### Bioinformatic Analysis

**Figure 1:**
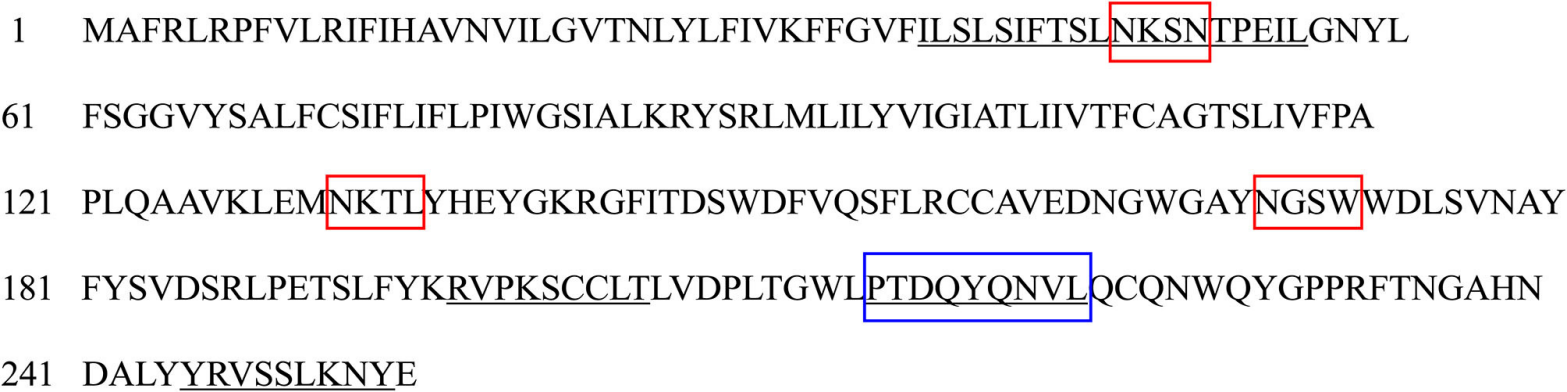
The deduced amino acids sequence of TSP11. Underlined: five protein kinase phosphorylation sites; border marked on frame: three potential N-terminal glycosylation sites; shaded: one tyrosine kinase phosphorylation site.

**Figure 2:**
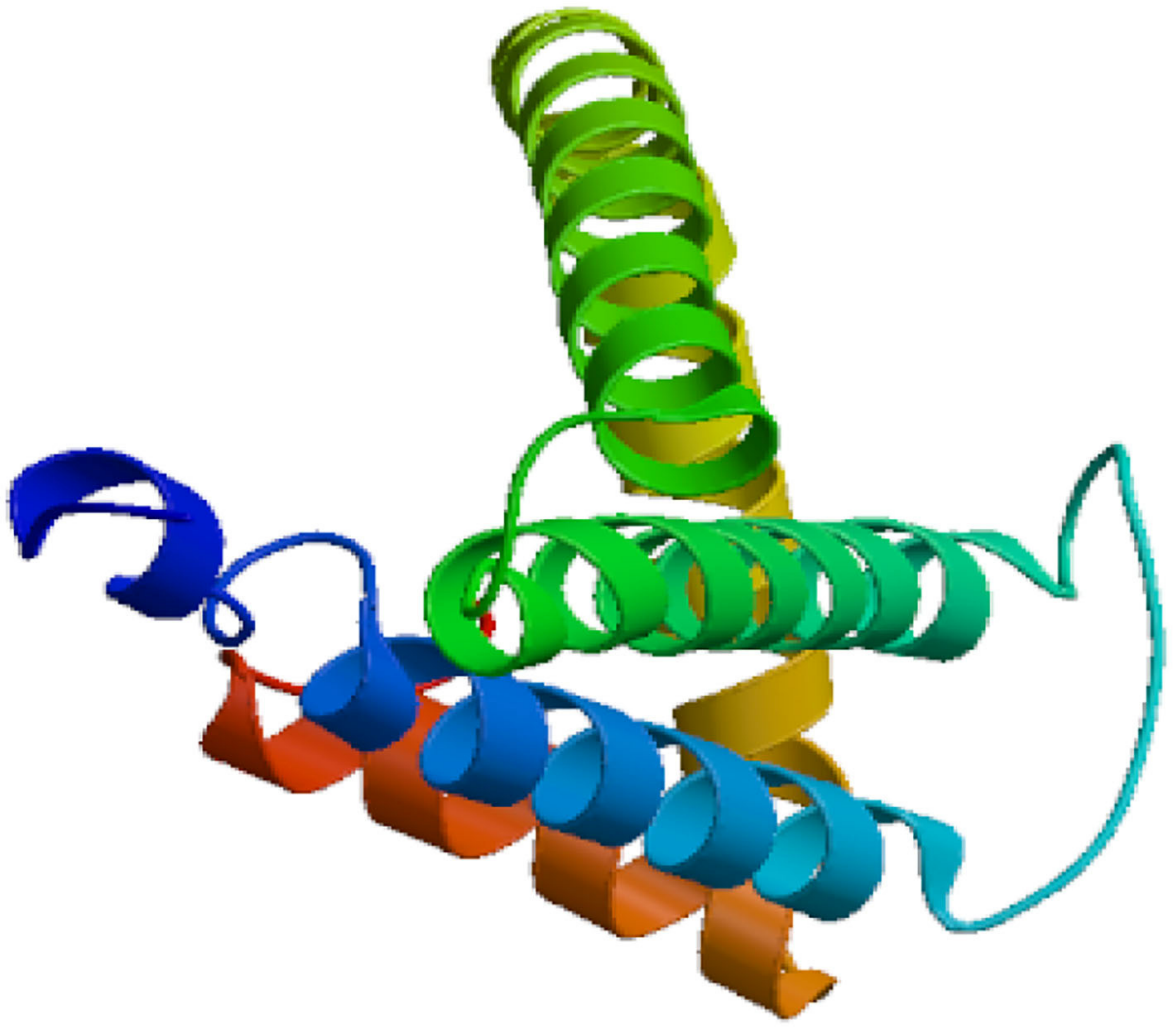
Predicted secondary and three-dimensional structure of the Eg-TSP11 protein.

**Figure 3:**
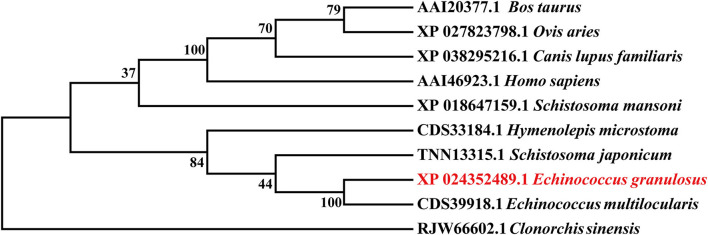
Neighbor Joining phylogenetic tree constructed using Eg-TSP11 and TSP proteins from other species.

The Eg-TSP11 cDNA sequence was comprised of 765 nucleotides, which encoded a putative protein of 254 amino acids (aa). TSP11 had a predicted pI of 8.91, a predicted mass of 27 kDa, three potential N-terminal glycosylation sites, five potential protein kinase phosphorylation sites, and one predicted tyrosine kinase phosphorylation site (Figure 1), including the three typical transmembrane regions (17–39, 59–81, and 94–116). TSP11 contains a NET-5-LIKE-LEL (the largest extracellular loop) located at amino acids 116–120 aa. TSP11 comprises seven predicted B cell linear epitopes (ep1: 48–55 aa; ep2: 123–123 aa; ep3: 143–143 aa; ep4: 162–170 aa; ep5: 186–189 aa; ep6: 210–219 aa; and ep7: 227–240 aa). The three-dimensional structure of TSP11 (judged by structural modeling) revealed multiple helices and folding regions, and its three-dimensional structure was mainly comprised of irregular coils (Figure 2). In the phylogenetic analysis, Eg-TSP11 was similar to TSP proteins from *Echinococcus multilocularis* (Figure 3).

#### Expression, Purification, and Recognition of rEg-TSP11

**Figure 4:**
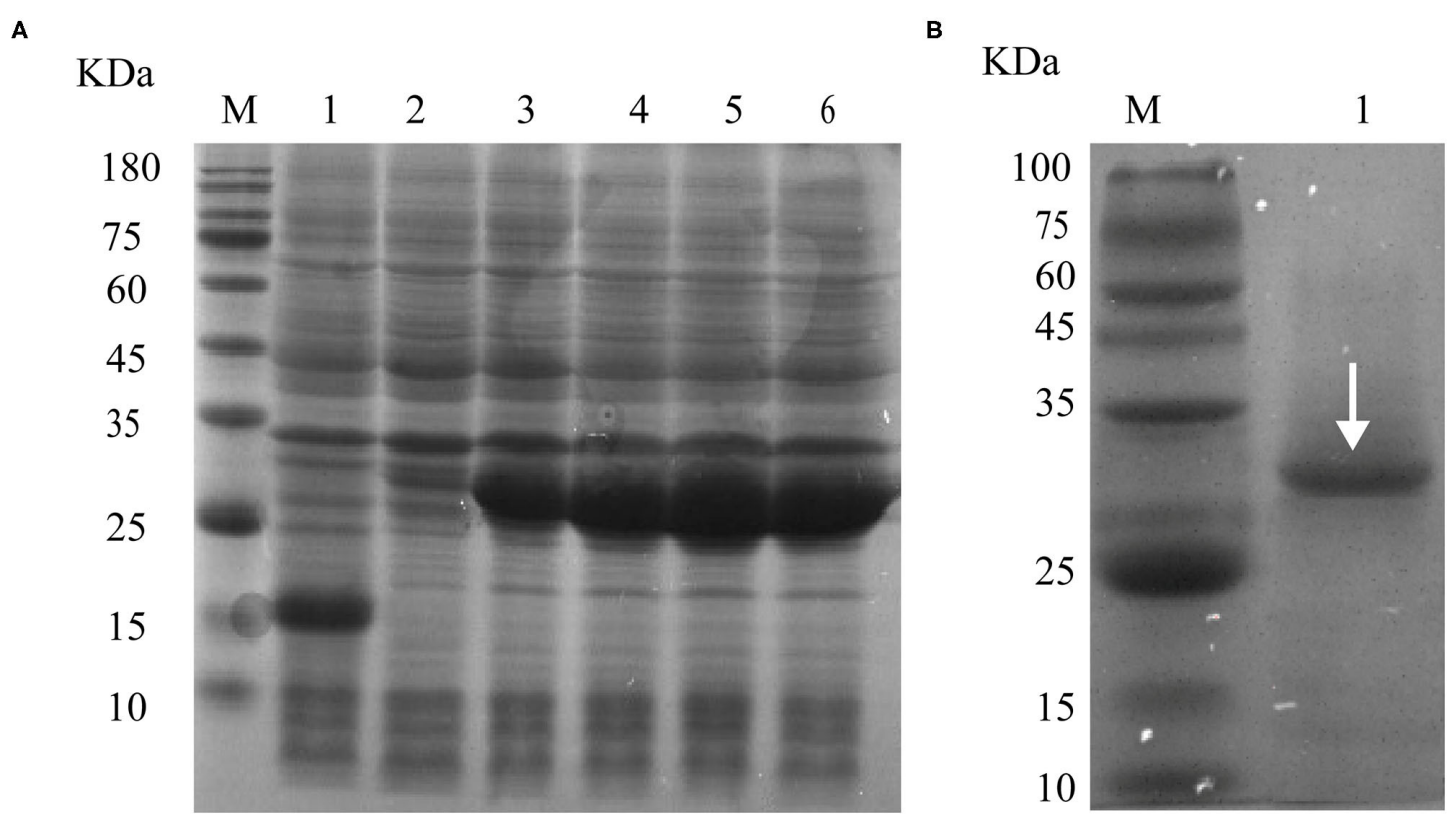
rEg-TSP11 expression and purification. **(A)** Lane M: Protein molecular weight markers; Lane 1, The recombinant plasmid was transferred into *E. coli* BL21 (DE3) followed by IPTG induction of pET-32a (+) empty vector for 6 h. Lane 2–6: Recombinant plasmids were transferred into *E. coli* BL21 (DE3) followed by IPTG induction of pET32a-TSP11 for 0, 2, 4, 6, and 8 h. **(B)** Lane M: Protein molecular weight markers. Lane 1: protein sample following purification of the His-tagged rTSP11 protein using an Ni^2+^ column.

The cDNA encoding the LEL of Eg-TSP11 was PCR-amplified from PSCs, and the recombinant rEg-TSP11 was expressed successfully. The purified rEg-TSP11 protein, which included a His-tag, showed a single band close to the predicted size of 32 kDa on 10% SDS-PAGE (Figure 4).

#### Western Blotting

**Figure 5:**
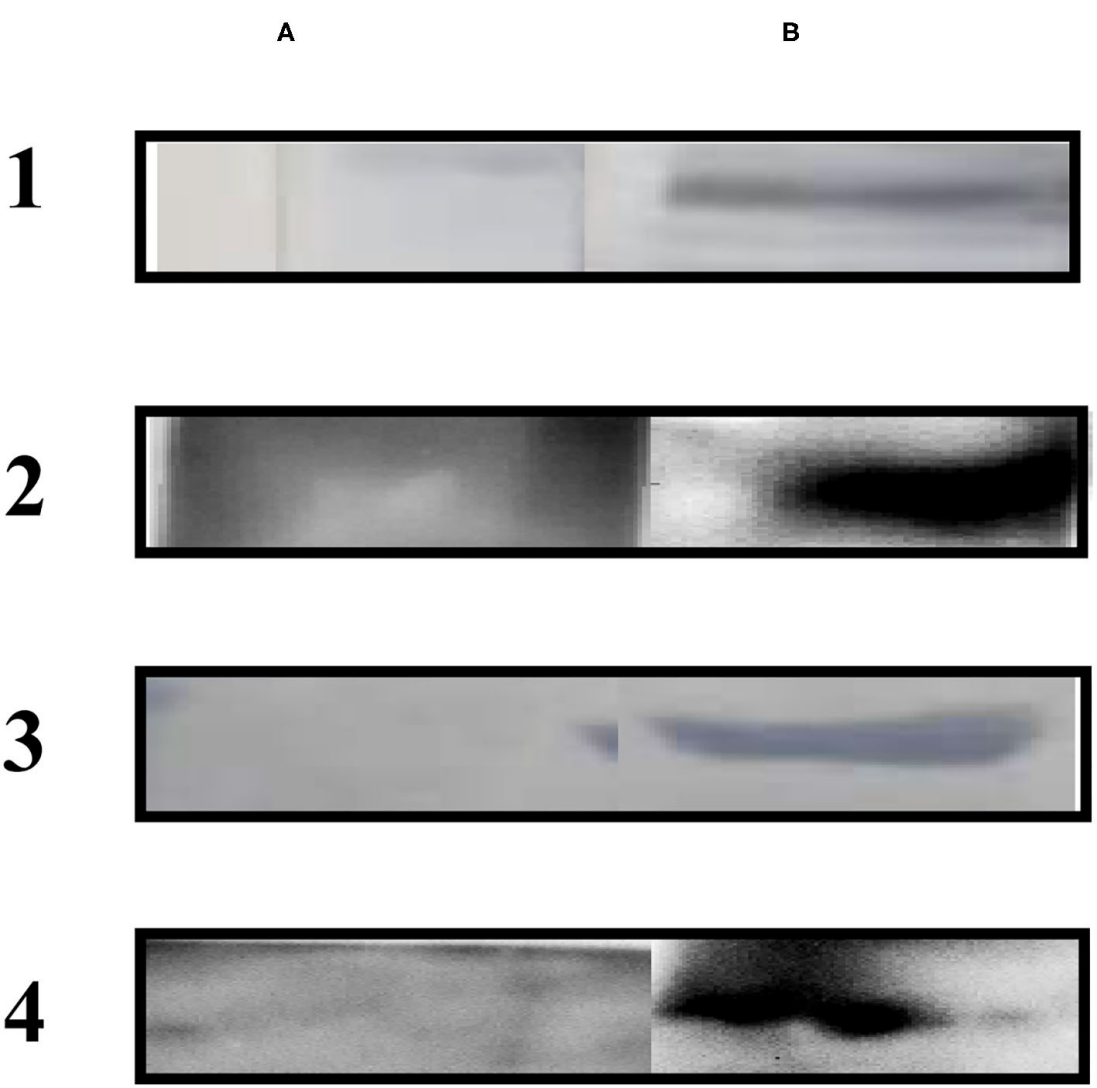
Western blotting analysis. **(A)** Lane A1: total protein extracts of PSCs probed with pre-immunized dog sera; Lane A2: total protein extracts of PSCs probed with pre-immunized dog sera; Lane A3: non-infected dog sera was used to probe purified rEg-TSP11; Lane A4: non-infected dog sera was used to probe purified rEg-actin. **(B)** Lane B1: total protein extracts of PSCs probed using anti-rEg-TSP11 dog sera; Lane B2: total protein extracts of PSCs probed using anti-rEg-actin dog sera; Lane B3: purified rEg-TSP11 probed with the serum of *E. granulosus* infected dog; Lane B4: purified rEg-actin probed with the serum of *E. granulosus* infected dog.

Immunoblotting using sera from dogs experimentally infected with *E. granulosus* showed a single ~32 kDa band. The native Eg-TSP11 protein from PSC extract could be recognized using anti-rEg-TSP11 dog IgG, with an apparent molecular mass of ~27 kDa, as expected. As anticipated, when reacted with pre-immunized dog serum or non-infected dog serum, no signal was observed with rEg-TSP11 (Figure 5).

#### qRT-PCR Analysis of Eg-TSP11 Expression

**Figure 6:**
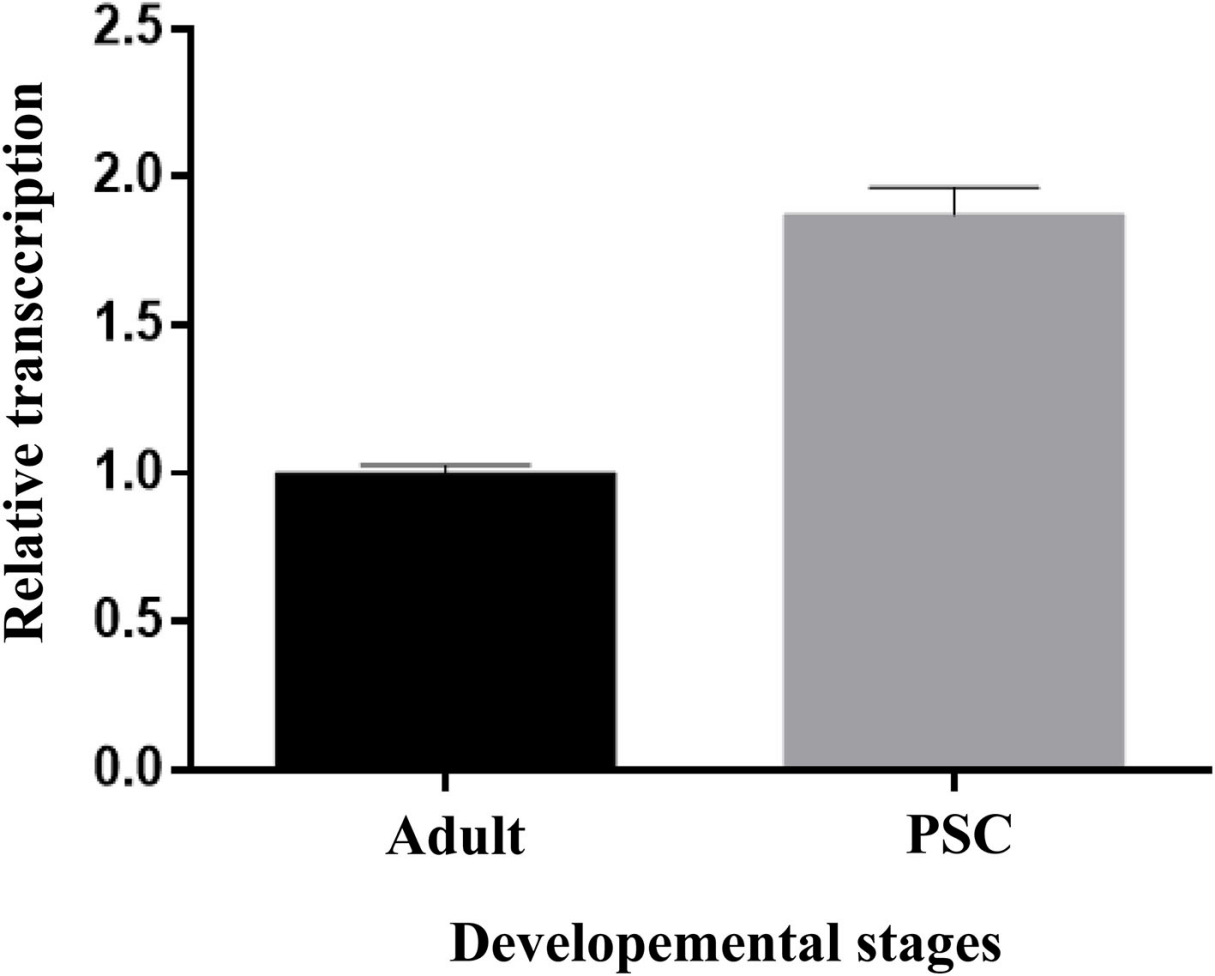
Expression analysis of Eg-TSP11 mRNA in different developmental stages of *E. granulosus*.

The relative transcription of Eg-TSP11 was assessed using qRT-PCR. Eg-TSP11 mRNA was detected in *E. granulosus* adult and protoscolex stages, with a significantly higher level in the PSC stage (*P* > 0.05; Figure 6).

#### Eg-TSP11 Localization During Different Life Stages of *E. granulosus*

**Figure 7:**
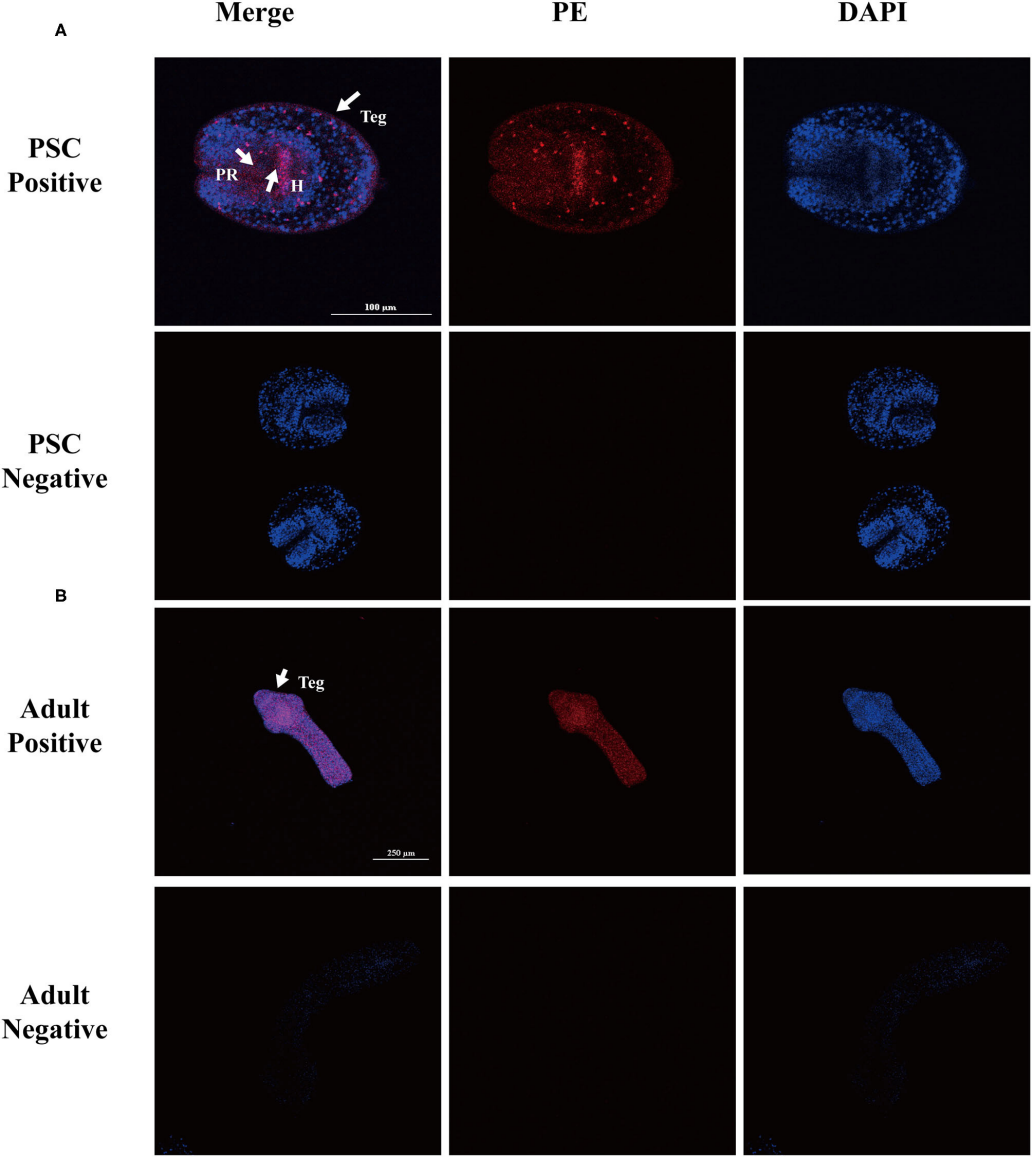
Immunofluorescent localization of Eg-TSP11 in different stages of *E. granulosus*. Eg-TSP11 was localized in the protoscolex and adult worm using specific anti-rEg-TSP11 IgG (positive) or pre-immune serum (negative), respectively. The nuclear DNA was stained with DAPI (blue). Teg, tegument; PR, parenchymal region; H, hook; *Scale bars*: 50 μm **(A)**, 250 μm **(B)**.

The localization of native Eg-TSP11 in PSCs and adult worms was determined by immunofluorescence using specific polyclonal antibodies against rEg-TSP11. In PSCs, the fluorescence signals were mainly localized in the tegument tissues and hook, while weak signals were detected in the parenchymal region (Figure 7A). In adult worms, weak signals were detected in tegument tissue (Figure 7B). No fluorescence signals were detected in the negative control samples.

#### Indirect ELISA

**Figure 8:**
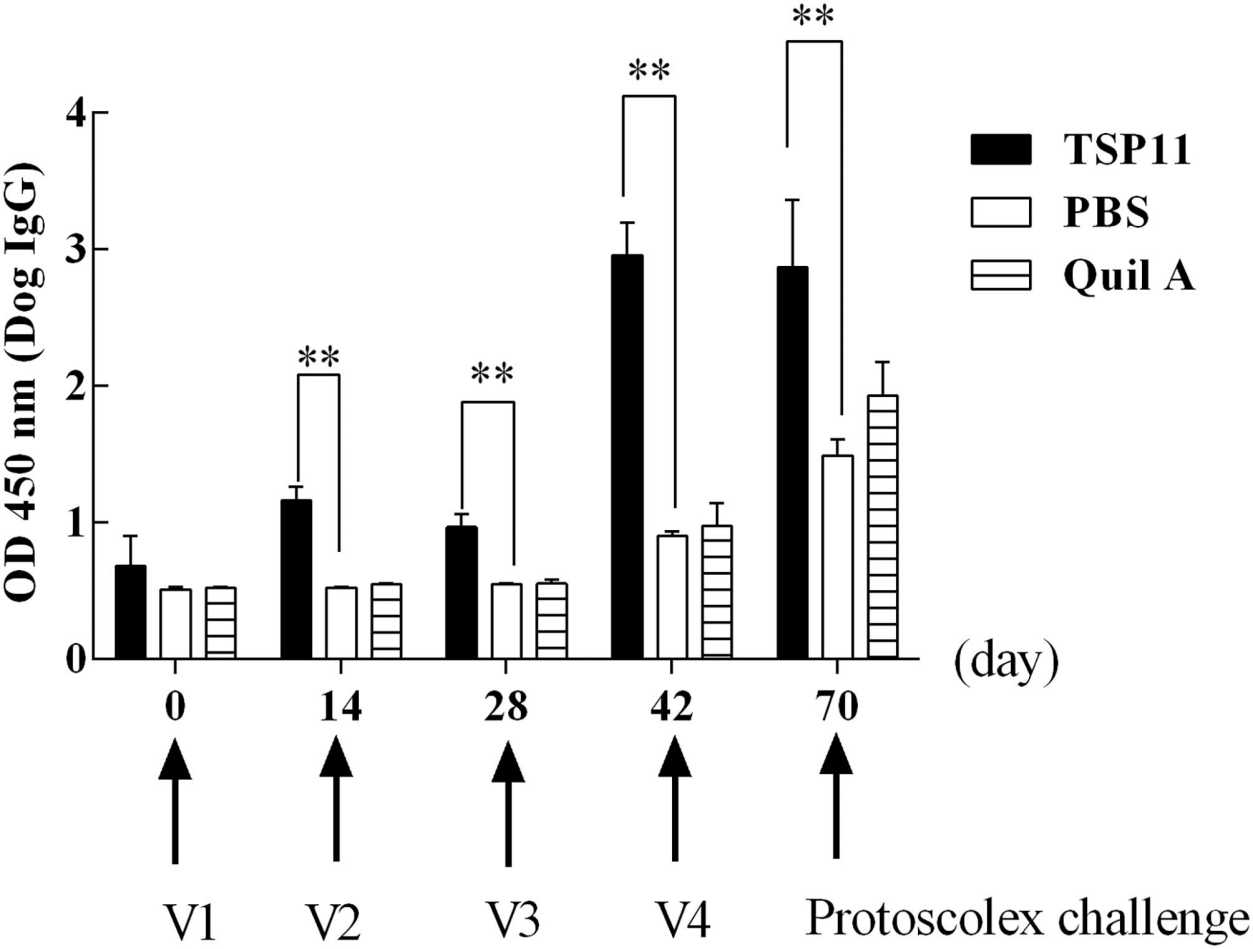
Changes in antibody IgG levels in dogs vaccinated with rTSP11+Quil A, Quil A alone, or PBS. ^*^P < 0.05; ^**^P < 0.01.

Indirect ELISA was used for a preliminary assessment of serum IgG changes after vaccination with rEg-TSP11. At 0, 14, 28, and 42 days after the first vaccination, sera from nine immunized dogs were tested. In the dogs immunized with rEg-TSP11, the IgG level increased significantly at 14, 28, and 42 days after immunization (14 d *t* = 11.35 *P* = 0.0003, 28 d *t* = 7.700, *P* = 0.00151, 42 d *t* = 14.89, *P* = 0.0001) and at 28 days post-challenge compared with the PBS control group (*t* = 4.729, *P* = 0.0091). IgG levels peaked 42 days post-vaccination (Figure 8).

#### Response of Canine Cytokines Induced by rEg-TSP11

**Figure 9:**
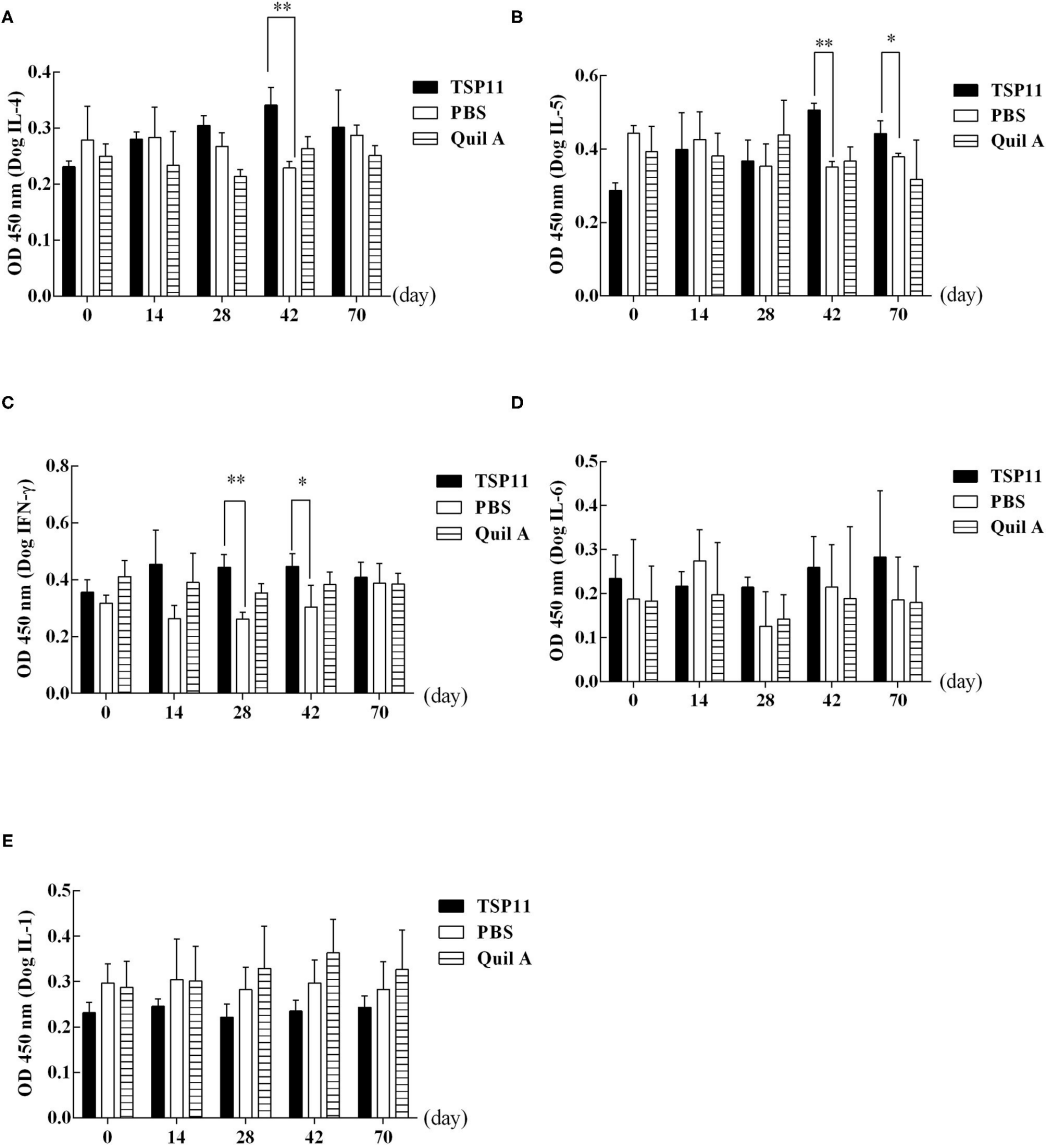
ELISA detection of the levels of different cytokines in dogs vaccinated with rTSP11+Quil A, Quil A alone, or PBS. **(A)** IL-4, **(B)** IL-5, **(C)** IFN-γ, **(D)** IL-6, E: IL-1. ^*^P < 0.05; ^**^P < 0.01.

ELISA was used to detect cytokines IFN-γ, IL-6, IL-5, and IL-4 at 0, 14, 28, and 42 days after immunization and at 28 days post-PSC challenge (Figure 9). Compared with that in the PBS control group, the serum IL-4 level increased significantly in the rEg-TSP11 vaccinated dogs at 42 days after immunization (14 d *t* = 0.09306 *P* = 0.9303, 28 d *t* = 2.129, *P* = 0.1003, 42 d *t* = 5.786, *P* = 0.0045); however, there was no significant change at day 28 post-PSC challenge (*t* = 0.3628, *P* = 0.7351) (Figure 9A). Moreover, the serum IL-5 level increased significantly at 42 days after immunization (14 d *t* = 0.3599 *P* = 0.7372, 28 d *t* = 0.3042, *P* = 0.7761, 42 d *t* = 11.08, *P* = 0.0004) and 28 days post-PSC challenge (*t* = 3.104, *P* = 0.0361) in the rEg-TSP11 vaccinated dogs (Figure 9B). Furthermore, the serum IFN-γ level increased significantly at 28 days and 42 days after immunization (14 d *t* = 2.592 *P* = 0.0605, 28 d *t* = 6.200, *P* = 0.0034, 42 d *t* = 2.803, *P* = 0.0487). There were no significant changes at day 28 post-PSC challenge (*t* = 0.4121, *P* = 0.7014) in the rEg-TSP11 vaccinated dogs (Figure 9C). In addition, there were no significant differences in serum IL-6 (Figure 9D) and IL-1 (Figure 9E) levels in rEg-TSP11 vaccinated dogs in the different groups.

#### Vaccine Efficacy of rEg-TSP11 Combined With Quil A

Vaccination using rEg-TSP11 mixed with Quil A resulted in a 76.80% reduction in the number of worms compared to that in the PBS control group (Table 1). This suggested that vaccination with rEg-TSP11 mixed with Quil A induced good protective efficacy (inhibition of worm growth) in Beagles against *E. granulosus* infection 28 days after PSC challenge.

**Table 1:** Number of Worms in vaccinated dogs after PSC challenge.

**Group (protein)**	**Dog ID number**	**Number of worms**	**Reduction %^**a**^**
PBS	1	13,200	
	2	4,800	
	3	5,700	
Average		7,900	-
Quil A	4	28,200	
	5	4,200	
	6	4,800	
Average		12,400	
Eg-TSP11	7	1,200	
	8	1,400	
	9	2,900	
Average		1,833	76.80
*P*-value^b^			0.100

a *Reduction% = [(average number of worms in the control group—the average number of worms in the experimental group)/(average number of worms in the control group)] × 100%*.

b *The Mann-Whitney U test was used to compare the worm burden median; P-value < 0.05 indicates that the reduction is significant between PBS (control) and experimental groups using median analysis*.

#### Worm Development in rEg-TSP11 Vaccinated Dogs

The results showed that in the rEg-TSP11-vaccinated group, the development and maturation of the worms was inhibited by 77.04%. Thus, vaccination with rEg-TSP11 induced a significant protective effect, as indicated by the inhibition of worm growth (Table 2).

**Table 2:** Worm segment development in the experimental groups.

**Group (protein)**	**Dog ID**	**Worm development**		
		**≤3 segments**	**≥4 segments**	**Worm inhibition**
Eg-TSP11	1	26	4	
	2	22	8	
	3	28	2	
Average		25	5	77.04%
PBS	4	9	21	
	5	13	17	
	6	7	23	
Average		9	21	–
Quil A	7	7	23	
	8	9	21	
	9	3	12	
Average		7	23	–

## Discussion

Tetraspanins are widely distributed in eukaryotes and participate in many cellular biological processes (22). They play a significant downregulatory role, especially in immune interactions with host molecules, including the major histocompatibility complex (MHC) (23, 24). This immune interaction is vital in the process of parasite immune evasion. The downregulation of the host immune response by tetraspanin allows the parasite to hide its non-self-state and evade detection by the host, leading to successful host infection. Therefore, targeting tetraspanins using a variety of methods, including RNA interference or monoclonal antibodies, could lead to the development of anti-parasite vaccines. Certain members of the tetraspanin family have been targeted in schistosomiasis (25–27), Clonorchiasis sinensis (28), and alveolar echinococcosis (29, 30), and as candidate vaccines for filariasis (31). However, there are few reports on the TSPs of *E. granulosus*. In this study, molecular cloning, prokaryotic expression, western blotting, and fluorescence immunolocalization were carried out. The immune protective effect of the rEg-TSP11 in dogs was evaluated by assessing the changes in antibody levels, the worm reduction rate, the inhibition of worm segmentation, and Th1/Th2 cytokine levels. These results provide the basis for determining the antigenic potential of rEg-TSP11.

The results of bioinformatic analysis showed that TSP11 has five protein kinase phosphorylation sites and one tyrosine kinase phosphorylation site, indicating that phosphorylation of TSP11 plays a pivotal role in *E. granulosus* growth and development (32). Specific epitopes determine the specificity of antigens (33). Therefore, epitope prediction is particularly important in the study of protein structure and function, and for the diagnosis of diseases and the design of vaccines (34). The present study found that there were seven potential B-cell epitopes in Eg-TSP11, among which the random coil regions were 227–240 (amino acid sequence: WQYGPPRFTNGAHN) and 210–219 (amino acid sequence: LTGWPTDQYQ). The results showed that these two regions had strong antigenicity, and most of the epitopes were located in the extracellular loop of tetraspanin LEL, which has a highly conserved cysteine sequence. Studies have shown that the LELs of some TSPs have good immunogenicity and reactivity and are potential vaccine candidate antigens (8); therefore, in the present study, we cloned and expressed the LEL of Eg-TSP11. This analysis indicated that Eg-TSP11 has good antigenic potential and provides a theoretical basis for Eg-TSP11 as a candidate antigen for the development of a vaccine against *E. granulosus* infection. Transcriptional analysis indicated that *Eg-TSP11* is expressed in both adult worms and the protoscolex, suggesting that Eg-TSP11 might play a role in the parasite life cycle. Moreover, we found that Eg-TSP11 was mainly expressed in the epidermis of adult worms and protoscolex. Interestingly, we also found that the serum from dogs with *E. granulosus* infection also recognized rEg-TSP11. In platyhelminths, the tetraspanin families are expanded and are likely to be components of the host-pathogen interface (35). Studies have shown that the tetraspanins could be part of extracellular vesicles that are released by helminths within hosts (36), that they bind to the Fc domain of host antibodies (37), and that they are highly immunogenic (38). Based on these findings, we speculated that Eg-TSP11 might be a component of the host-pathogen interface and could be part of the extracellular vesicles released by *E. granulosus* within hosts.

Quil A, as a saponin adjuvant, is widely used in veterinary vaccines and human and veterinary immunology. Saponins can induce strong adjuvant responses to T cell-dependent antigens (39). Saponins can also induce a strong cytotoxic CD8^+^ lymphocyte reaction and enhance the response to mucosal antigens (40), and when combined with cholesterol and phospholipids, immune stimulating complexes are formed (41). The Quil A adjuvant can activate cellular and humoral immune responses against a variety of viruses, bacteria, parasites, and tumor antigens (41, 42). Hence, in this study, we used rEg-TSP11 mixed with Quil A to induce a more effective immune response. Indirect ELISA was used to detect changes in IgG levels in dog serum. The results showed that rEg-TSP11 could significantly stimulate the body to produce specific antibodies.

Echinococcosis is a zoonotic parasitic disease that is also an immune imbalance disease (42). *E. granulosus* infection initiates long-term interactions with the host. In this interaction, the existence of Th1/Th2 type immune responses plays a crucial role. When the worm changes the immune balance of Th1 and Th2 in the host's own system via specific antigens, it produces inhibitory cytokines to escape the host's immune defense (42). Moorhead et al. reported that CD4^+^ T cytokines in mice can be divided into two subgroups. Th1 type cells mainly secrete IL-1, IFN-γ, and TNF-β, and Th2 cells mainly secrete IL-4, IL-5, and IL-10 (43). A previous study found that in patients with echinococcosis, Th2 type cytokines were mainly increased in their serum (44). Another study showed that the Th1-mediated cellular immune response can enhance the host's anti-infection ability in the early stage of echinococcosis, playing an important role in the control of the initial infection of echinococcosis (45). In the later stages of infection, the main humoral immune response is mediated by Th2 type immunity (45). It has also been suggested that the transformation from a Th1 immune response to a Th2 type immune response is beneficial for the growth and development of *E. granulosus*, such that IL-4, IL-5, IFN-γ, and other cytokines play an important role in the immune interaction between the host and *E. granulosus* (45). In the present study, rEg-TSP11 induced Th1 and Th2 immune responses in dogs, and the changes in IFN-γ cytokine levels were more obvious in the pre-immune period. However, after challenge with PSCs for 28 days, marked changes in IL-5 cytokine levels were observed. Such delayed cytokine responses to PSC Ags have been previously described in infected mice and dogs (20, 46), which indicated that the humoral immune response mediated by Th2 type immunity is the main pathway in the late stage of echinococcosis.

We vaccinated dogs with Eg-TSP11 mixed with Quil A, and used PBS as a control. In terms of worm reduction rates, the rEg-TSP11 immunized group showed a worm reduction of 76.80% compared with that in the PBS control group, representing a significant reduction in the worm burden. Moreover, the rEg-TSP11 immunized group showed a development and maturation inhibition of the worms was 77.04%. However, 70.00 and 76.67% of worms developed to the adult worms in the PBS control groups and Quil A, while only 22.96% of worms developed to the adult worms in the rEg-TSP11 immunized group. This results also suggested that the rEg-TSP11 vaccine induced significant protective efficacy in terms of inhibition of worm growth compared with the control dogs. This was better than the oral vaccine comprising a live carrier of Salmonella EgA31 EGTRP (70–80%), although individual differences were large (21), and slightly lower than that of EgM123 (89.2%) (19). Thus, rEg-TSP11 showed good performance in decreasing worm burden and inhibiting segment development. For humans, cattle, sheep, and other intermediate hosts, the threat comes from the eggs of adult *E. granulosus*. If a vaccine can be developed to reduce the number of parasites and inhibit their development by immunizing the definitive host, we might effectively reduce the amount of ovulation and reduce the threat. In this study, rEg-TSP11 showed good performance in terms of the worm reduction rate and the inhibition of segmental development, and thus is expected to be a good vaccine candidate antigen for the definitive host of *E. granulosus*.

## Conclusions

An increasing number of studies are focusing on the TSP because of its wide distribution and important biological roles in a variety of organisms. In the present study, we cloned and prokaryotically expressed Eg-TSP11. Furthermore, we evaluated the immunoprotective effects of rEg-TSP11 in model dogs. The results indicated that rEg-TSP11 could induce the production of specific antibodies in dogs and significantly increase or decrease the levels of Th1 and Th2 cytokines. In addition, rEg-TSP11 decreased worm burden and inhibited segment development. These findings revealed that rEg-TSP11 might be a potential candidate vaccine antigen against *E. granulosus* infection in dogs.

## Data Availability Statement

The original contributions presented in the study are included in the article/supplementary material, further inquiries can be directed to the corresponding author/s.

## Ethics Statement

The animal study was reviewed and approved by the Care and Use of Laboratory Animals of the Xinjiang Academy of Agricultural and Reclamation Sciences (Shihezi, China) (Approval No. 2019-012, April 9, 2019). All animals were handled in strict accordance with the animal protection laws of the People's Republic of China (a draft animal protection law was released on September 18, 2009) and the National Standards for Laboratory Animals in China (executed on January 5, 2002).

## Author Contributions

JX, PZ, and NW performed the experiments, collected and analyzed the data, and prepared the manuscript. WW and XM contributed to study design and implementation. YZ and JM collected the parasite specimens and performed immunofluorescence experiments. ZW and XB conceived the study, participated in its design, coordinated the project, and contributed to the interpretation of the results. All authors contributed to the article and approved the submitted version.

## Funding

This research was funded by the Important Science & Technology Specific Projects of State Key Laboratory of Sheep Genetic Improvement and Healthy Production (grant number 2021ZD02), the National Natural Science Foundation of China (grant number 31860701), and the International Scientific and Technological Cooperation Projects of Xinjiang Production and Construction Corps (grant numbers 2021BC008 and 2020BC007).

## Conflict of Interest

The authors declare that the research was conducted in the absence of any commercial or financial relationships that could be construed as a potential conflict of interest.

## Publisher's Note

All claims expressed in this article are solely those of the authors and do not necessarily represent those of their affiliated organizations, or those of the publisher, the editors and the reviewers. Any product that may be evaluated in this article, or claim that may be made by its manufacturer, is not guaranteed or endorsed by the publisher.
